# Cyclophosphamide causes osteoporosis in C57BL/6 male mice: suppressive effects of cyclophosphamide on osteoblastogenesis and osteoclastogenesis

**DOI:** 10.18632/oncotarget.21000

**Published:** 2017-09-18

**Authors:** Dongfeng Zhao, Chenglong Wang, Yongjian Zhao, Bing Shu, Youji Jia, Shufen Liu, Hongshen Wang, Junli Chang, Weiwei Dai, Sheng Lu, Qi Shi, Yanping Yang, Yan Zhang, Yongjun Wang

**Affiliations:** ^1^ Longhua Hospital Affiliated to Shanghai University of Traditional Chinese Medicine, Shanghai, P.R. China; ^2^ Spine Disease Research Institute, Shanghai University of Traditional Chinese Medicine, Shanghai, P.R China; ^3^ Key Laboratory of Theory and Therapy of Muscles and Bones, Ministry of Education, Shanghai University of Traditional Chinese Medicine, Shanghai, P.R. China; ^4^ Central Laboratory of Research, Longhua Hospital, Shanghai, P.R. China; ^5^ School of Rehabilitation Science, Shanghai University of Traditional Chinese Medicine, Shanghai, P.R. China

**Keywords:** cyclophosphamide, chemotherapy, osteoporosis, osteoblastogenesis, osteoclastogenesis

## Abstract

The clinical evidence indicated that cyclophosphamide (CPD), one of the chemotherapy drugs, caused severe deteriorations in bones of cancer patients. However, the exact mechanisms by which CPD exerts effects on bone remodeling is not yet fully elucidated. Therefore, this study was performed to investigate the role and potential mechanism of CPD in osteoblastogenesis and osteoclastogenesis. Here it was found that CPD treatment (100mg/kg/day) for 7 days led to osteoporosis phenotype in male mice. CPD inhibited osteoblastogenesis as shown by decreasing the number and differentiation of bone mesenchymal stem cells (MSCs) and reducing the formation and activity of osteoblasts. Moreover, CPD suppressed the osteoclastogenesis mediated by receptor activator for nuclear factor-κ B ligand (RANKL) as shown by reducing the maturation and activity of osteoclasts. At the molecular level, CPD exerted inhibitory effect on the expression of components (Cyclin D1, β-catenin, Wnt 1, Wnt10b) of Wnt/β-catenin signaling pathway in MSCs and osteoblasts-specific factors (alkaline phosphatase, Runx2, and osteocalcin). CPD also down-regulated the expression of the components (tumor necrosis factor receptor-associated factor 6, nuclear factor of activated T-cells cytoplasm 1, c-Fos and NF-κB) of RANKL signaling pathway and the factors (matrix metalloproteinase 9, cathepsin K, tartrate-resistant acid phosphates and carbonic anhydrase II) for osteoclastic activity. Taken together, this study demonstrated that the short-term treatment of CPD induced osteoporosis in mice and the underlying mechanism might be attributed to its marked suppression on osteoblastogenesis and osteoclastogenesis, especially the effect of CPD on bone formation might play a dominant role in its detrimental effects on bone remodeling.

## INTRODUCTION

Cancers cause severe mortality and lead to significant societal burdens globally. The chemotherapy represents one of the most effective strategies in treating types of cancers [1]. Cyclophosphamide (CPD), one class of drugs from alkylating agent family, increases the survival rate of cancer patients [2, 3] by inhibiting the proliferation of tumor cells via intercalating with DNA [4, 5]. However, CPD also causes severe side effects on the skeleton, leading to increased risks of osteoporosis [6, 7]. In addition, it was reported that the treatments with either CPD or CPD regiments induced the early onset of menopause in women, and consequently led to the occurrence of osteoporotic phenotype at both at trabecular bone and cortical bone [8, 9].

The available evidence demonstrated that the osteoporotic phenotype in cancer patients treated with CPD is caused, at least partially, by hypogonadism [6, 10], because CPD causes damages on ovaries in women and decreases levels of androgens in men [9–12]. The failure of ovaries leads to the decrease of estradiol (E2) production similar to that seen in postmenopausal osteoporosis [13]. Furthermore, CPD exerted inhibitory effect on bone formation by arresting the differentiations of preosteoblasts [14] and increased the level of follicle-stimulating hormone (FSH) and luteinizing hormone (LH) [15–17], which are inhibitors on bone formation. Additionally, several studies have reported that the CPD regiments enhanced the expression of bone resorption markers in long bones of female rats [18–20], promoted the differentiation of mesenchymal stem cells (MSC) to adipocytes [18, 21], and induced bone resorption by altering the ratio of receptor activator for nuclear factor-κ B ligand (RANKL)/osteoporotegerin (OPG) in serum of early-stage breast cancer patients [12]. Thus, CPD-induced osteoporosis may be attributed to its damages on sex gland and bone formation and resorption. However, the underlying mechanism for the effect of CPD on osteoblast and osteoclast is yet to be determined.

Wnt/β-catenin plays critical roles in regulating osteoblastogenesis [22] and bone formation [23]. Wnt family members, as such as Wnt1 and Wnt10b, are secreted cysteine-rich glycoproteins in osteoblast and interact with β-catenin to activate Wnt signaling [24, 25]. The activation of Wnt/β-catenin promotes the differentiation of osteoblast from mesenchymal stem cells (MSCs) by stimulating the expression of Cyclin D1 and c-Myc [23, 26, 27]. Glycogen synthase kinase 3β (Gsk-3β) plays crucial roles in regulating the activity of canonical Wnt signaling pathway [28]. Normal expression of Gsk-3β inhibits the combination of Wnt ligand to β-catenin through activating the phosphorylation of β-catenin [29]. In contrast, the stimulated expression of Gsk-3β increases the ubiquitination and proteosomal degradations of β-catenin thus to reduce the translocation of β-catenin into the nucleus and secondly inhibits osteoblast differentiation from MSCs [30, 31]. Furthermore, Wnt/β-catenin pathway works in collaborating with several osteoblast-related regulators, including ALP, Runx2, and osteocalcin [32, 33], which are necessary for osteoblastogenesis.

Osteoclasts differentiate from bone marrow macrophages (BMMs) upon stimulation by two essential cytokines, macrophage colony stimulating factor (M-CSF) and receptor activator for nuclear factor-κ B ligand (RANKL) [34]. RANKL binds to its receptor RANK, a member of the TNF receptor super family, to recruit TNF receptor-activated factors family members (TRAF-1, 2, 3, 4, 6) [35, 36]. The recruitments of TRAFs activates numerous signaling pathways (IκBα/NF-кB, ERK, JNK, p38) which are necessary for osteoclastogenesis [37, 38]. Meanwhile, RANKL also stimulates the expression of nuclear factor of activated T-cells-cytoplasm 1(NFATc1) [38] and c-Fos [39] as well as osteoclast genes, such as those encoding matrix metalloproteinase 9 (MMP9), cathepsin K (Ctsk), tartrate-resistant acid phosphates (TRAP) and carbonic anhydrase II (Car2) [40]. The biological influences of CPD on the osteoclastogenesis are not clearly known, even though some studies indicated that CPD suppressed bone resorption by arresting the proliferation of osteoclasts [6] and the combination chemotherapy with CPD, epirubicin and 5-fluorouracil stimulated the RANKL-mediated osteoclatsogenesis [18]. Therefore, it is necessary to investigate the exact roles of CPD in regulating RANKL-mediated osteoclastogenesis.

The earlier studies demonstrated that the anti-tumor effects of CPD were caused by its regulation on Wnt/β-catenin pathway [41, 42]. One recent study found that CPD exerted anti-tumor effects in sarcoma S180 tumor-bearing mice by down-regulating the expression Wnt/β-catenin and Cyclin D1 [41]. Additionally, CPD exerted inhibitory effects on angiogenesis in Lewis lung cancer mice through negatively modulating the Wnt/β-catenin signaling pathway [42]. To our knowledge, the direct effects of CPD on Wnt/β-catenin during the process of osteoblastogenesis are not yet investigated [6, 43].

This study sought to investigate the effects of CPD on bones *in vivo* using mouse models. Moreover, *in vitro* assays were performed to investigate the effects of CPD on the differentiation and activity of osteoblasts and osteoclasts. Finally, *in vitro* mechanistic studies were carried out to elucidated the mechanism by which CPD exerts its effects on osteoblast and osteoclast differentiation.

## RESULTS

### Identification of 4-OH-CPD in conditioned medium (CM)-CPD working solution

**Figure 1:**
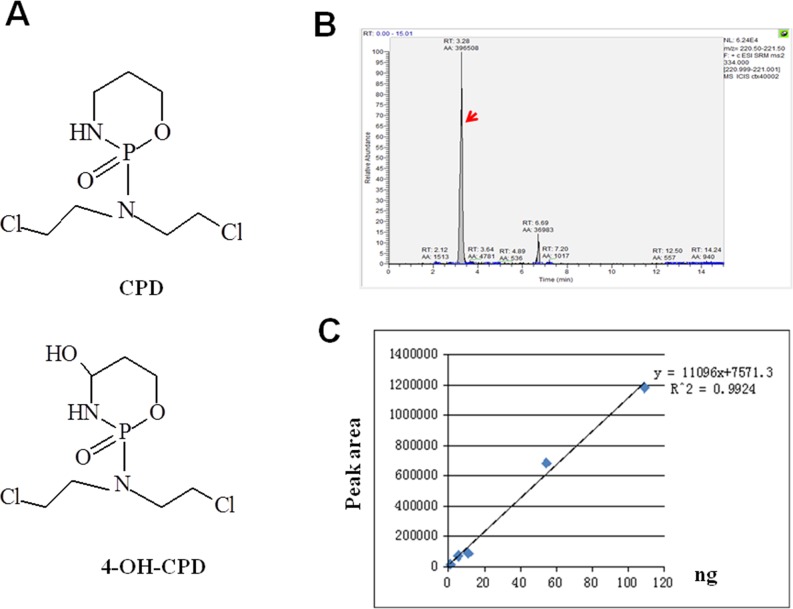
The identification of conditioned medium (CM)-CPD working solution **(A)** Chemical structure of cyclophosphamide (CPD) and its medial metabolic product (4-OH-CPD) that can be detected in the conditioned medium working solution. **(B)** The LC/MS/MS chromatograms of 4-OH-CPD (as indicated by the arrow) in CPD-H (4000μM) in multiple reaction monitoring (MRM) mode. **(C)** Standard curve were made with 10 times serial dilution of 4-Hydroperoxy CPD.

The structures of CPD and 4-OH-CPD are shown in Figure 1A. 4-OH-CPD is the active intermediate metabolite. The concentration of 4-OH-CPD is usually used as one critical marker to monitor the efficacy of CPD-based chemotherapy and therefore the liquid chromatography mass spectrometry (LC-MS/MS) analysis were carried out. Briefly, the concentration of 4-OH-CPD in CPD-H (4000 μM) was detected in multiple reactions monitoring (MRM) mode. The peak area (Figure 1B) represented the quality or concentration of 4-OH-CPD. The calculated concentration of 4-OH-CPD in CPD-H (4000μM) was about 175.3 ng/mL (0.6 μM) based on the standard curve (Figure 1C). This study identified and quantified the active 4-OH-CPD in conditioned medium (CM)-CPD working solution, which confirmed and guaranteed the biological influences of CPD in the following *in vitro* studies.

### CPD treatment induced the phenotype of osteoporosis

Numerous clinical observations have demonstrated that the chemotherapy treatment with CPD caused osteoporosis phenotypes. This study was conducted to firstly confirm whether CPD exerts similar effects in mice. Male C57BL/6 mice were treated with CPD (100 mg/kg/d, i.p.) or saline as control for 7 consecutive days. The mice were sacrificed at 3, 7 and 12 d after CPD injection. To exclude the systematic toxicity, the changes of body weight were measured during experimental period. The body weights of CPD-treated mice were significantly decreased (*P* < 0.05) at 3d and 7d during the CPD treatment period. While, the body weight at 12d in CPD group restored to the same level as that in the saline control group (Supplementary Figure 1). Moreover, the animal experiments with three independent times were performed (12 mice/group) and no mice were dead during the experiment. Therefore, CPD did not induce systemic toxicity in this study.

**Figure 2:**
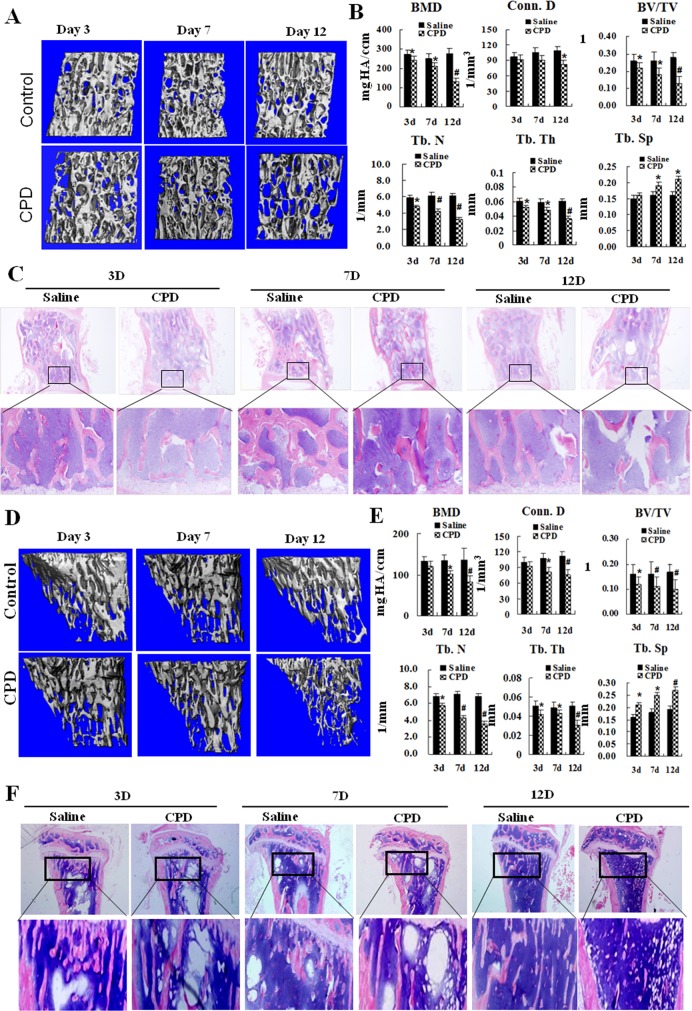
The treatment with cyclophosphamide caused severe bone loss in mice **(A)** Male C57BL/6 mice were administered with saline or cyclophosphamide (CPD, 100mg/kg/days (d)) for 7 consecutive days by intraperitoneal injections. The mice were maintained for additional 3,7and 12 days before harvesting. Micro-CT analysis was performed on lumbar vertebrate (LV1~3) and one representative imagine from each group was shown. **(B)** The bone parameters were shown as the following: Bone mineral density (BMD); Connectivity density (Conn. D); Bone volume over total volume (BV/TV); Trabecular bone number (Tb.N); Trabecular bone separation (Tb. Sp); Trabecular bone thickness (Tb. Th). **(C)** H&E stains were performed on LV1~3. **(D)** One representative image on the proximal metaphysis of tibia was shown. **(E)** The bone parameters were shown as the following: Bone mineral density (BMD); Connectivitydensity (Conn. D); Bone volume over total volume (BV/TV); Trabecular bone number (Tb. N); Trabecular bone separation (Tb. Sp); Trabecular bone thickness (Tb. Th). **(F)** H&E stains were performed on tibias. Values were expressed as means ± SEM. ^*^ *P* < 0.05, ^#^*P* < 0.01, vs. Saline control group at the same time point.

Lumbar spine (LV1~3) and proximal end of tibia measurement were scanned by micro-CT to obtain skeletal phenotype following the instruction of the ASBMR guideline in 2010 [44]. The result indicates that CPD-intervention caused significant osteoporosis phenotypes both at lumbar spine (LV1~3) (Figure 2A) and proximal end of tibia (Figure 2D). Compared to saline control group, the application of the CPD caused significant decreases in bone mineral density (BMD), connectivity density (Conn. D), trabecular bone number (Tb. N) and trabecular bone thickness (Tb. Th) of LV1~3 (Figure 2B) at day 3 (*P* < 0.05), 7 (*P* < 0.05) and 12 (*P* < 0.01). In addition, CPD treatment resulted in the decrease of BMD, Conn. D, trabecular bone volume over total volume (BV/TV), Tb. N and Tb. Th as well as the increase of trabecular bone separation (Tb. Sp). Moreover, these alterations of trabecular bone parameters were most significant (*P* < 0.01, Figure 2E) at day 12. The changes of these quantitative parameters of trabecular bone at LV1~3 and tibia were in accordance with the histological images by H&E staining (Figure 2C and 2F) which clearly showed the loss of trabecular bone number and network. Thus, CPD treatment induced the severe osteoporotic phenotype at trabecular bone of male mice.

### CPD down-regulated the expression of osteoblast differentiation-related factors

**Figure 3:**
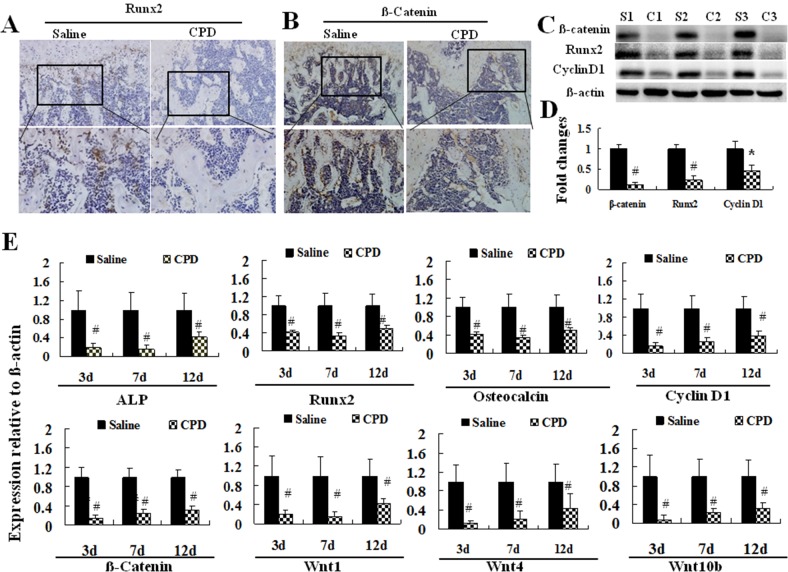
Cyclophosphamide exerted inhibitory effects on the expression of osteoblast differentiation-related factors The male C57BL/6 mice were administered with saline or cyclophosphamide (CPD, 100mg/kg/d) for 7 consecutive days by intraperitoneal injection. The tibia was harvested, decalcified and fixed to make paraffin sections after the mice were maintained for additional 12 days. The protein expression of Runx2 **(A)** and β-catenin **(B)** were assessed by immunohistological (IHC) staining. **(C, D)** LV4~5 from CPD-treated or saline control mice, randomly at least 3 mice each group, were harvested from at 12 days after intervention of CPD. The protein expressions of β-catenin, Runx2, and Cyclin D1 were assessed with western blot (C) and quantified. (D), ^*^ *P < 0.05*, ^#^ *P* < 0.01, vs. saline. S:saline, C:CPD. **(E)** Real-time PCR was performed to assess the mRNA expression of Runx2, β-catenin, Osteocalcin, alkaline phosphatase (ALP), Wnt1 and Cyclin D1 with mRNA from LV4~5. Values were expressed as means ± SEM. **^#^** *P* < 0.01, vs. saline control group. At least 3 mice in each group were performed for IHC assays, and Real-time PCR assays.

To explore the effects of CPD on the expression of a series of osteoblast differential-related biomarkers, we first detected the protein expression of Runx2 (Figure 3A) and β-catenin (Figure 3B) in tibia of CPD-treated mice at day 12. Meanwhile, the protein expression of β-catenin, Cyclin D1, and Runx2 were assessed with LV4~5 from CPD-treated or saline control mice (Figure 3C and 3D). Next, the mRNA expression of Runx2, β-catenin, Osteocalcin, ALP, Wnt 1, Wnt4, Wnt10b and Cyclin D1 in lumbar vertebra (Figure 3E). CPD treatment largely decreased the positive signals of Runx2 and β-catenin as well as markedly reduced the mRNA expression of Runx2, ALP, β-catenin, Osteocalcin, Wnt 1, Wnt4, Wnt10b and Cyclin D1 of LV4~5 (*P* < 0.01).

### CPD treatment decreased the number of bone mesenchymal stem cells

**Figure 4:**
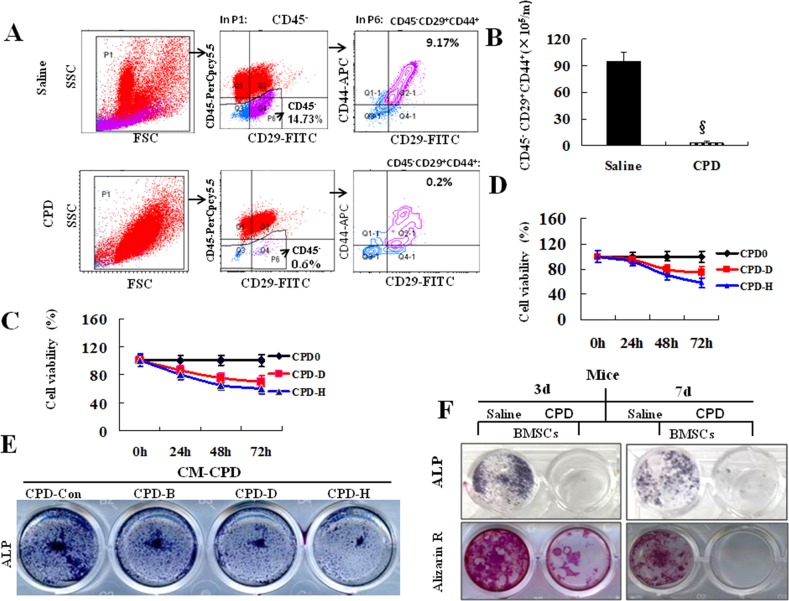
Cyclophosphamide decreased the number of mesenchymal stem cells, cell viability and capability of osteoblastogenesis **(A)** Bone mesenchymal stem cells (MSCs) from tibia/femur in saline or CPD-treated male mice were prepared. The expressions of positive CD29^+^, CD44^+^, and CD45^-^ were tested by the fluorescence activating cell sorter (FACS). **(B)** The number of MSCs from per mice(m). Primary bone MSCs **(C)** or C3H10T1/2 lineages **(D)** were treated with CPD-Con, CPD-D or CPD-H working solutions for 0, 24, 48 and 72 hours before measuring cell viability. **(E)** Primary bone MSCs, isolated from the bilateral femur/tibia of male C57BL/6 mice, was cultured with CPD-Con, CPD-B, CPD-D and CPD-H for 2 days. All the assays were performed alkaline phosphatase (ALP) staining after 7 days’ treatment with osteoblast differentiation introduction medium. **(F)** Primary bone MSCs, isolated from the bilateral femur/tibia from 3d or 7d after the final injection of saline - or CPD-treatment. Then, primary bone MSCs was cultured with osteoblast differentiation introduction medium for 7 days for ALP staining and 21 days for Alizarin R stains. All the *in vitro* assays were independently repeated for 3 times or with at least 3 mice.

Bone primary mesenchymal stem cells were isolated and flushed with α-MEM from the bilateral femur and tibia of naïve, saline control or CPD-treated male C57BL/6 mice. Mature osteoblasts were derived from bone marrow mesenchymal stem cells, the number of which was determined and compared in CPD-treated or saline control mice. We next fully examined the effects of CPD on the number of MSCs by assessing the expression of CD29^+^, CD44^+^, and CD45^-^, which are critical markers for MSCs [45]. The percentage of CD45^-^ is 14.73% in saline control mice but is 0.6% in CPD-treated mice. In addition, the percentage of CD29^+^CD44^+^CD45^-^ is 9.17% in saline control and is 0.2% in CPD-treated group (Figure 4A). Furthermore, statistical analysis indicates that the number of MSCs per CPD-treated mice is much lower than saline control (Figure 4B).

### CPD inhibited cell viability and osteoblastic differentiation of MSC

Osteoblasts differentiate from bone MSCs. Normal viability of MSCs and osteoblastic differentiation are requirements for BMD and bone function. We further investigated the roles of CPD in cell viability of MSCs and osteoblastogenesis. MSCs from male C57/BL mice (Figure 4C) and C3H10T1/2 cell lineage (Figure 4D) were treated with different concentrations of CPD working solutions (CPD-Con, CPD-D and CPD-H) to measure the cell viability. CPD dose-dependently decreased the cell viability of primary bone MSCs and C3H10T1/2 cells and the most significant suppressive effect was observed at time point of 72 hours.

The differentiation of MSCs to osteoblasts plays a key role in regulating bone formation. Bone MSCs were treated with CPD-Con, CPD-B, CPD-D and CPD-H, and then stained for ALP. The results showed that CPD dose-dependently inhibited the amount of ALP-positive staining in bone MSCs (Figure 4E). Meanwhile, both Alizarin Red (Figure 4F, above panel) and ALP staining (Figure 4F, bellow panel) demonstrated that there were decreased osteoblastogenesis of bone MSCs, collected at day 3 and day 7, in CPD-treated mice.

### CPD suppressed osteoblastogenesis by down-regulating Wnt/β-catenin pathway

**Figure 5:**
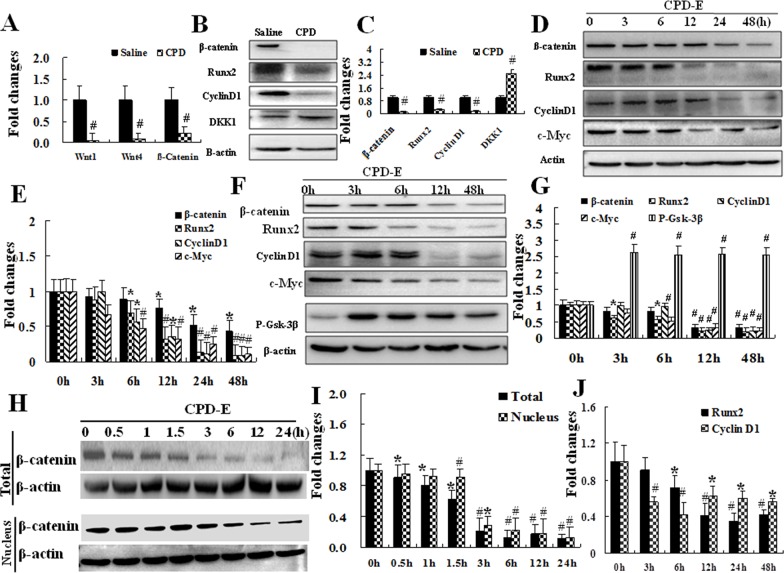
Cyclophosphamide suppressed the activation of Wnt/ß-catenin pathway involved in osteoblastogenesis in mesenchymal stem cells **(A)** LV4~5 from saline or CPD- treated mice (7d) were harvested before accessing the mRNA expression of Wnt1, Wnt4, and β-catenin by real-time PCR. ^#^*P* <0.01, vs. saline group. Primary bone MSCs from saline or CPD-treated mice (7d) were harvested, cultured, and lysed 1days later. The expressions of β-catenin, Runx2, Cyclin D1and DKK1 were measured with western blot assessments **(B)** and quantified **(C)**. ^#^*P* <0.01, ^§^<0.001 vs. saline group. **(D, E)** Primary bone MSCs from CPD-treated or saline control were harvested and cultured with CPD-H for 0, 3, 6, 12, 24 and 48h. The protein expressions of β-catenin, Runx2, Cyclin D1 and c-Myc were assessed with western blot (D) and quantified (E), ^*^ *P < 0.05*, ^#^ *P* < 0.01, vs. 0 hour. (F and G) C3H10T1/2 was cultured with CPD-Con or CPD-H for 0h, 3h, 6h, 12h and 48h. The protein expressions of ß-catenin, Runx2, Cyclin D1, c-Myc and P-Gsk-3β were assessed with western blot **(F)** and quantified **(G)**. ^*^ *P* < 0.05, ^#^ *P* < 0.01, vs. 0 hour. (H&I) Total and nucleus proteins were harvest from C3H10T1/2 and the proteins were harvested at different time (0, 0.5, 1, 1.5, 3, 6, 12, 24 h). The protein expression of β-catenin was assessed with western blot **(H)** and quantified **(I)**. ^*^ *P* < 0.05, ^#^ *P* < 0.01, vs. 0 hour.C3H10T1/2 cell was treated with CPD-H for different times (0, 3, 6, 12, 24, 48h). The mRNA expression of Runx2 and Cyclin D1 was assessed with real-time PCR **(J)**. ^*^ *P* < 0.05, ^#^ *P* < 0.01, vs. 0 hour. For quantification in each assays, Ratios of targeted markers to β-actin were obtained by dividing the densitomentic reading of these markers with that of β-actin, and the value calculated at 0h was set as 1.00. The All the *in vitro* assays were independently repeated for 3 times or with at least 3 mice.

Wnt/β-catenin pathway plays important roles in stimulating osteoblastogenesis. The activity of Wnt/β-catenin signaling was investigated (Figure 5). The real-time PCR result showed that CPD significantly suppressed mRNA levels of Wnt1, Wnt4, and β-catenin in LV4~5 of CPD-treated mice as compared to those of saline control group (*P* < 0.01, Figure 5A). Furthermore, primary bone MSCs were isolated from saline- or CPD-treated (100 mg/kg) mice, and the western blot showed that the protein expression of active β-catenin, Runx2, and Cyclin D1 was significantly decreased, and DDk1 protein expression was markedly increased in CPD-treated group (Figure 5B and 5C) Next, we further investigated the effects of CPD exerts on a series of markers during osteoblastogenesis at different time points (0,3,6,12,24,48h) in primary bone MSCs, such as the expressions of active β-catenin, Runx2, and Cyclin D1 and c-Myc. Our finding indicates that CPD significantly inhibits the expression of β-catenin, Runx2, Cyclin D1 and c-Myc (Figure 5D and 5E).

Furthermore, we investigated whether CPD exerts inhibitory effects on the expression of total and nuclear β-catenin. C3H10T1/2, one common MSCs cell line, were treated with CPD and the total and nuclear proteins were assessed by western blot analysis. Our result showed that CPD both exerts inhibitory effects on the expression of both total and nuclear β-catenin (Figure 5H and 5I). Next, C3H10T1/2 was used to assess the expression of β-catenin activated signaling in osteoblastogenesis. The result shows that CPD significantly suppressed the expression of active β-catenin, Runx2, c-Myc, and Cyclin D1; however, CPD stimulates the expression of phospho-Gsk-3β (Figure 5F and 5G), which are critical markers for osteoblastogenesis. In addition, we also determined the expression of Runx2 and Cyclin D1 with RNA by C3H10T1/2. To our expectation, CPD inhibited the expressions of Runx2 and Cyclin D1 in a time-dependent manner (Figure 5J).

### CPD decreased the number of TRAP-positive mature osteoclasts *in vitro* osteoclast assays

Bone remodeling is modulated by the tight coupling between osteoblast mediated bone formation and osteoclasts mediated bone resorption. Since CPD exerted inhibitory effects on osteoblastogenesis, it was interesting to investigate the effects of CPD on osteoclastogenesis and bone resorption.

**Figure 6:**
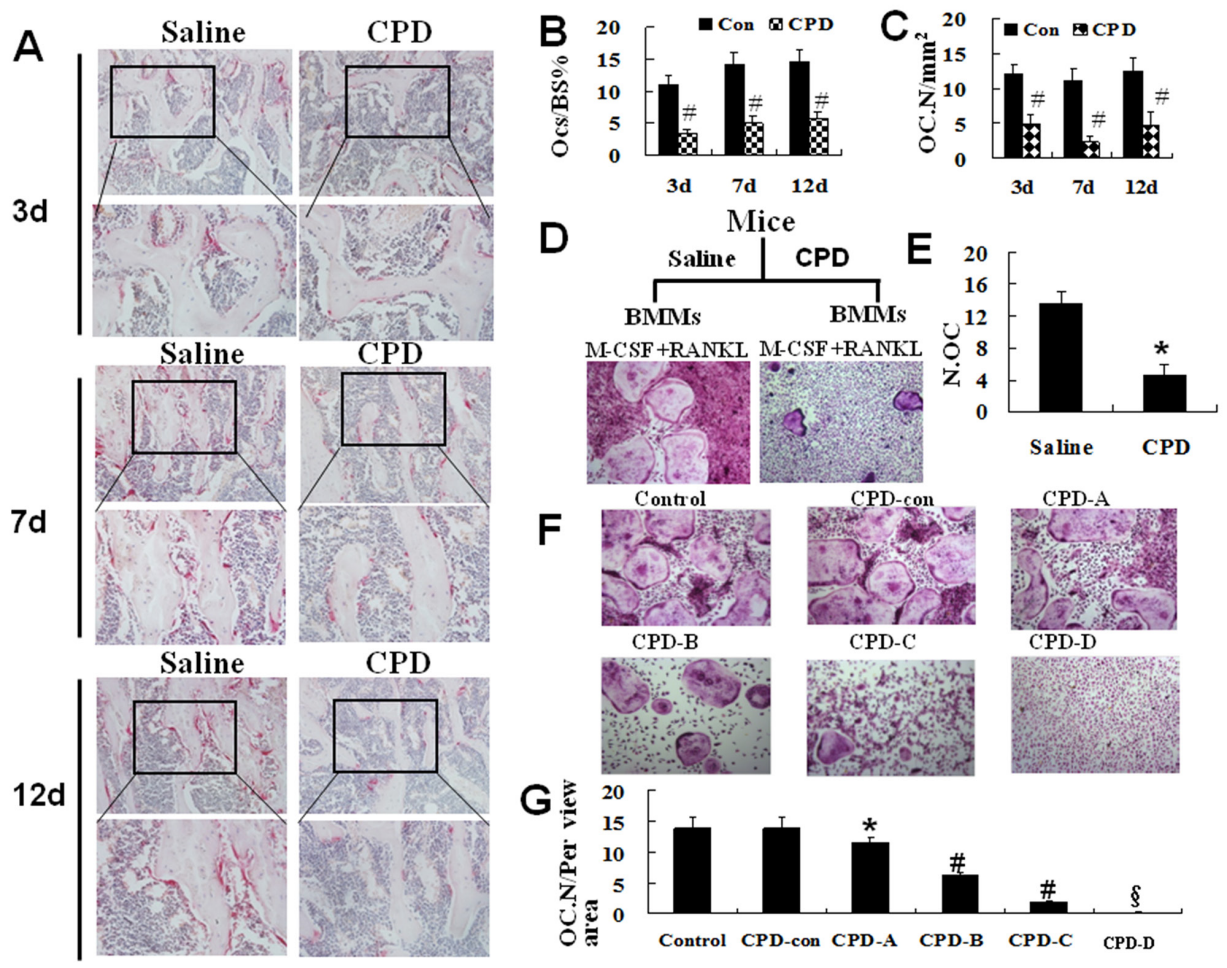
Cyclophosphamide suppressed the activity of TRAP and the capacity of osteoclastogenesis in mice **(A)** TRAP staining was performed on femurs at day 3, 7 and 12 after the C57BL/6 mice were injected with saline (Con) or cyclophosphamide (CPD) for 7 consecutive days. **(B)** Quantification of osteoclasts-covered surface over bone surface. **(C)** Osteoclasts number. **(D)** BMMs from saline or CPD-treated mice (12 days) were cultured with M-CSF (44 ng/ml) and RANKL (100 ng/ml) for 5 days before performing TRAP staining. **(E)** The number of multinucleated TRAP-positive cells (>3 nuclei).^*^ *P* < 0.01, vs. saline control. **(F)** BMMs from C57BL/6 mice were harvested, and treated with M-CSF (44 ng/ml) and RANKL (100 ng/ml) as control, or M-CSF (44 ng/ml) and RANKL (100 ng/ml) plus CPD-Con, CPD-A, CPD-B, CPD-C and CPD-H for 5 days before performing the TRAP staining. The number of osteoclast were quantified **(G)**, ^*^ *P* <0.05, ^#^ *P* < 0.01, ^§^ *P* < 0.001, vs. saline control.

Male C57BL/6 mice were treated with CPD (100 mg/kg/d, i.p.) or saline as control for 7 consecutive days. Subsequently, the mice were sacrificed at 3, 7 and 12 d after CPD treatment. TRAP activity in distal femoral metaphysis (Figure 6A) was less observed in CPD-treated mice at 3, 7 and 12 d as compared to those of saline control, and the CPD treatment significantly reduced the number of osteoclasts-covered surface (Figure 6B) and osteoclasts number (Figure 6C) in femur. These findings indicated that the osteoclastic resorptive activity was greatly depressed in CPD-treated mice.

To further confirm the inhibitory effect of CPD on RANKL-mediated osteoclastogenesis, we next carried out *ex vivo* assays with Bone marrow macrophages (BMMs, namely osteoclast precursors) from CPD-treated mice or saline control. M-CSF and RANKL are two essential and sufficient factors for osteoclastogenesis [34]. Primary BMMs were treated with M-CSF (44 ng/ml) and RANKL (100 ng/ml) for 5 days before performing TRAP staining (Figure 6D). BMMs from saline control differentiated to TRAP-positive osteoclasts, but few from CPD-treated mice (*P* < 0.01, Figure 6E), indicating that CPD-treatment impaired osteoclast differentiation.

### CPD dose-dependently inhibited osteoclastogenesis

Next, osteoclastogenesis assays were performed with BMMs from wide-type C57BL/6 mice. BMMs that treated with M-CSF and RANKL as positive control. Meanwhile, BMMs were treated with M-CSF and RANKL plus CPD-Con, or CPD-A, or CPD-B, or CPD-C or CPD-D for 5 days before performing TRAP staining. As the results shown in Figure 6F, both positive control and CPD-Con formed numerous osteoclast. In contrast, there were fewer osteoclasts existed in CPD-treated group and no osteoclasts were observed in CPD-D (2000 μM, *P* < 0.001). These results indicated that CPD dose-dependently inhibited RANKL-mediated osteoclastogenesis.

To select the proper concentration of CPD, we further assessed the viability of BMMs in the presence of CPD working solutions without RANKL to exclude the cytotoxicy of CPD. BMMs were treated in the presence of M-CSF alone plus each CPD working solutions (Control, CPD-Con, CPD-A to CPD-H) for 5 days before performing cell viability assays with cell counting kit (CCK-8). However, there were no significance of the percentages of viable cells between each CPD working solutions group and control (Supplementary Figure 2). Taken together, these findings indicated that CPD dose-dependently inhibited RANKL-mediated osteoclastogenesis without affecting the viability of BMMs.

### CPD inhibited the expression of TRAF-6, NFATc1 and c-Fos in mice or in BMMs

RANKL binds to its receptor RANK in the membrane of pre-osteoclasts, and recruits members of TRAF family, such as TRAF-3 and TRAF-6 [35], to activate a series of downstream signaling, such as the action of critical transcription factors (NFATc1, c-Fos) that are necessary for osteoclastogenesis [37]. To identify the molecular mechanism involved in the regulation of CPD on osteoclastogenesis, the expression of TRAF-3, TRAF-6, NFATc1 and c-Fos was measured.

**Figure 7:**
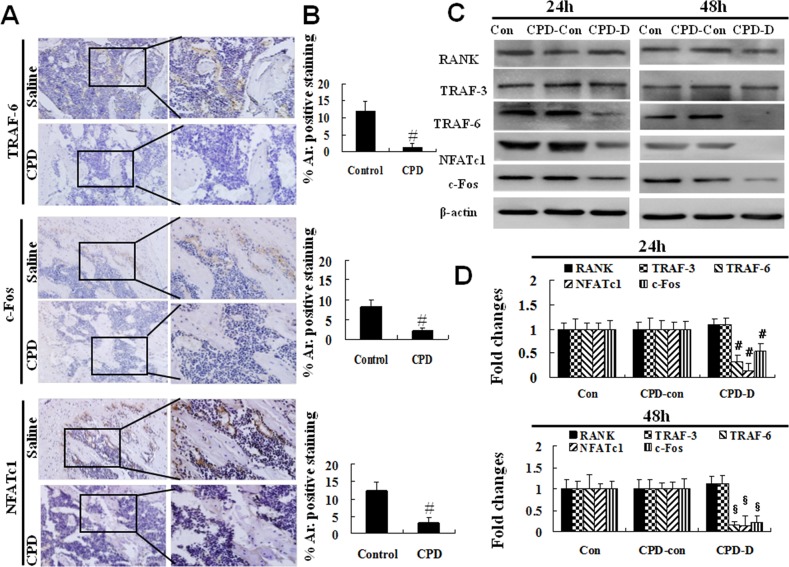
Cyclophosphamide caused the decreased expression of TRAF-6, c-Fos, NFATc1 in mice or in BMMs **(A)** 4-6-week-old male C57BL/6 mice were administered with saline or cyclophosphamide (CPD, 100 mg/kg/day) for 7 consecutive days by intraperitoneal injections. Mice were then maintained for additional 12 days before harvesting. The protein expression of TRAF-6, NFATc1 and c-Fos in tibia were assessed by immunohistochemistry. One set of representative images (20× and 40×) were shown, and the percentage (%) of the positive staining area to the whole view area were quantified **(B)**. ^#^ *P* < 0.01, vs. saline control. **(C)** BMMs were treated with M-CSF (44 ng/ml, Con), or plus CPD-Con or CPD-D for 24h or 48h. The protein expression of RANK, TRAF-3, TRAF-6, NFATc1 and c-Fos were measured. **(D)** Ratios of RANK, TRAF-3, TRAF-6, NFATc1 and c-Fos to β-actin were obtained by dividing the densitomentic reading of RANK, TRAF-3, TRAF-6, NFATc1, and c-Fos with that of β-actin, and the value calculated for con at 24h was set as 1.00. The protein bands were quantified. ^#^*P* < 0.01, ^§^*P* < 0.001 vs. control.

As compared to those in control group, the protein expression of TRAF-6, NFATc1 and c-Fos were significantly decreased in the tibia of mice at day 12 after CPD treatment (*P* < 0.001, Figure 7A and 7B). However, there was no significant difference in the expression of TRAF-3 and RANK between the two groups (Data not shown).

Furthermore, the suppressive effect of CPD on transcription factors for osteoclastogenesis was further assessed using primary BMMs treated with CPD for 24 h and 48 h. The protein expression of TRAF-6, NFATc1 and c-Fos was significantly down-regulated at both 24 h (*P* < 0.01) and 48 h (*P* < 0.001) as compared to those of CPD-Con group (Figure 7C), while, CPD did not affect the protein expression of RANK and TRAF-3 in BMMs (Figure 7D).

### CPD repressed RANKL-activated activation of NF-κB, while exerting no inhibitory effect on the activation of ERK, JNK and p38

**Figure 8:**
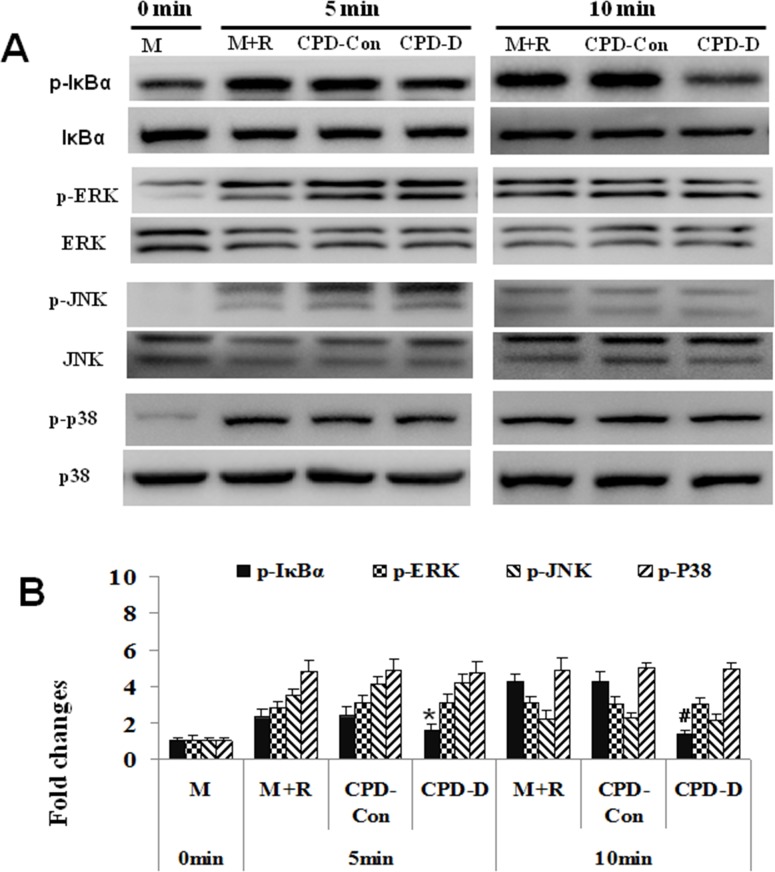
CPD repressed RANKL-activated expressions of NF-κB, while exerting no inhibitory effect on the expressions of ERK, JNK and p38 **(A)** BMMs were cultured with M-CSF (44 ng/ml) alone (M), M-CSF (44 ng/ml) and RANKL (100 ng/ml) (M+R), M+R plus CPD-Con, or CPD-D for 5 or 10 min. The protein expression of non-phosphorylated and phosphorylated form of IκBα, ERK, JNK, and p38 was determined. Ratios of the phosphorylated form to the total protein were obtained by dividing the densitometric reading of the phosphorylated form with that of the total proteins in each group, and 0 min was set as 1.00, and quantified **(B)**, ^*^ *P* < 0.05, ^#^ *P* < 0.01, vs. control or 0 min.

To further elucidate the molecular mechanism by which CPD inhibits osteoclastogenesis, we next examined the protein expression of key factors involved in RANK signaling pathway, such as NF-κB, JNK, ERK and p38 [32]. The levels of phosphorylated form of IκBα, JNK, ERK and p38 were measured to determine the activation of NF-κB, JNK, ERK and p38 signaling pathway by using Western blot analysis (Figure 8A and 8B). The results showed that RANKL treatment (M+R) tremendously increased levels of phosphorylated expression of IκBα, JNK, ERK and p38 at 5 minute and 10 minute in BMMs comparing to control without RANKL treatment (M). Phosphorylation of JNK, ERK and p38 were not affected by CPD-Con or CPD-D. However, CPD-D suppressed the phosphorylation of IκBα in contrast to control or CPD-Con at 5min (*P* < 0.05) and 10 min (*P* < 0.01), indicating that CPD did not inhibit the activation of JNK, ERK or p38 protein, but suppressed the activation of NF-κB in BMMs.

### CPD inhibited the expression of osteoclastic resorptive factors activated by RANKL

**Figure 9:**
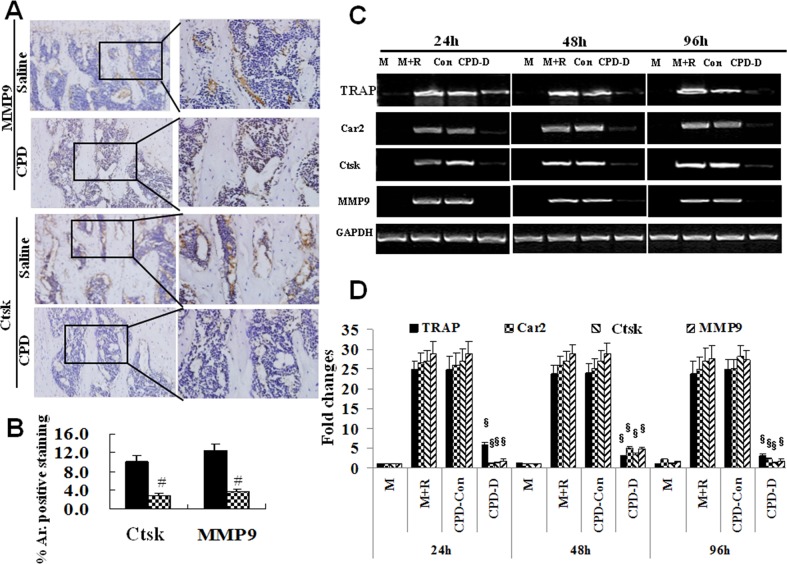
Cyclophosphamide caused the depressed expression of Ctsk and MMP9 in mice and Ctsk, MMP9, TRAP and Car II in BMMs **(A)** 4-6-week-old male C57BL/6 mice were administered with saline or cyclophosphamide (CPD, 100 mg/kg/day) for 7 consecutive days by intraperitoneal injections. The protein expression of Ctsk and MMP9 in tibia of mice at day 12 after CPD treatment was assessed. One set of representative images (20× and 40×) under each condition were shown, and the percentage (%) of the positive staining area to the whole view area were quantified **(B)** ^#^ *P* <0.01, vs. Saline control. **(C)** BMMs were treated with M-CSF (44 ng/ml, M), or combined with RANKL (100 ng/ml, M+R), or M+R plus CPD-Con, or CPD-D for 24h, 48h and 96h prior to isolation of total RNAs for semi quantitative RT-PCR analysis. **(D)** Quantification of the mRNA expression of TRAP, Car2, Ctsk and MMP9, ^§^ *P* < 0.01, vs. CPD-Con.

RANKL up-regulates the differential expression of numerous genes, which are not only requirement for normal osteoclastogenesis but also necessary for bone resorption [46]. Particularly, bone resorption activity was determined by a series of RANKL-stimulated resorptive factors, including MMP9, Car2, Ctsk and TRAP [40], the expression of which were measured in this study. The results demonstrated that the protein expression of MMP9 and Ctsk was significantly down-regulated in CPD-treated group as compared to those in saline control group (*P* < 0.05, Figure 9A and 9B).

To further confirm the inhibitory effect of CPD on RANKL-activated genes in osteoclastogenesis, we next performed *in vitro* investigations to assess mRNA levels of MMP9, Car2, Ctsk, and TRAP with CPD-D in BMMs. As shown in Figure 9C and 9D, RANKL significantly activated the gene expression of MMP9, Ctsk, TRAP and Car2 at 24, 48 or 96 h in BMMs. However, CPD-D significantly suppressed RANKL-induced expression of MMP9, Ctsk, TRAP and Car2 genes at 24, 48, and 96 h in BMMs. Our results demonstrated that CPD significantly inhibits RANKL-mediated expression of MMP9, Ctsk, TRAP, and Car2 genes in the process of osteoclast formation. Taken together, these findings indicated that CPD exerts inhibitory effects on RANKL-induced expression of osteoclast genes encoding MMP9, Ctsk, TRAP and Car2.

## DISCUSSION

**Figure 10:**
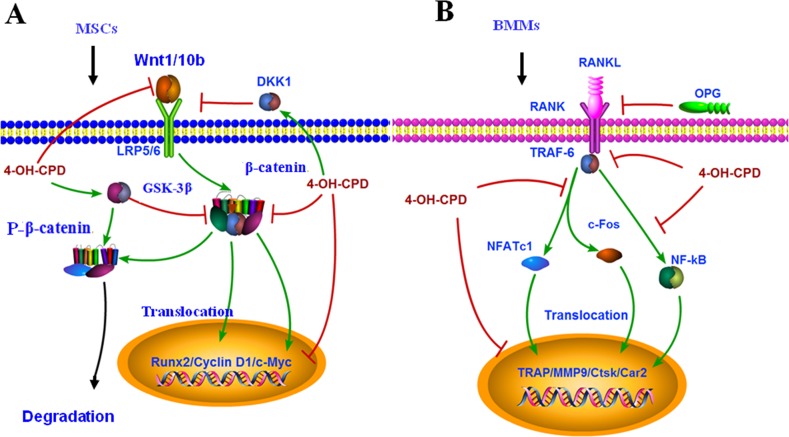
Molecular mechanism of cyclophosphamide in inhibiting osteoblastogensis and osteoclastogenesis **(A)** Cyclophosphamide (CPD) inhibits osteoblastogenesis by suppressing Wnt/β-catenin activated Cyclin D1, Runx2, c-Myc and stimulating the expression of GSK-3β and DKK1. **(B)** CPD inhibits osteoclastogenesis by suppressing RANKL-activated expression of TRAF-6, NFATc1, NF-кB, c-Fos and osteoclast encoding genes activity of MMP9, Ctsk, TRAP and Car2.

This is the first study to elucidate the roles of CPD in osteoblastogenesis and osteoclastogenesis using both *in vitro* and *in vivo* assays. Our study found that CPD treatment induced significant deteriorations of bones in mice. On one hand, CPD exerted the inhibitory effects on osteoblastogenesis through suppressing the Wnt/β-catenin signaling shown by the decreased expression of β-catenin, Wnt 1, Wnt10b and Cyclin D1 and the increased expression of DKK1. Consequently, CPD decreased the expression of osteoblast-specific factors like Runx2, Osteocalcin, ALP and the number of bone MSCs(Figure 10A). On the other hand, CPD inhibited the activity of osteoclast as demonstrated by the down-regulation of MMP9, Ctsk, TRAP and Car2. Moreover, RANKL-mediated osteoclastogenesis was also suppressed by CPD, which down-regulated the expression of TRAF-6, NFATc1, NF-κB and c-Fos (Figure 10B). Finally, the serum level of PINP, one of the bone formation markers, and β-CTX, one of the bone resorption markers were both decreased in CPD-treated mice (Data not shown). This study has provided new insights into the roles of CPD in bone remodeling, especially for understanding the underlying mechanism for bone loss of CPD-treated patients.

CPD, one member of alkylating agent family, was artificially synthesized in 1958 and prescribed in clinical practice from 1970s for treating a series of diseases [47], including cancers, immunological diseases, and rheumatoid arthritis. CPD exerts anti-tumors effect by suppressing the proliferation of tumor cells via inhibiting the replication of DNA [48] and serves as one of the most widely used chemotherapy drugs. The prescriptions of CPD regiments have greatly increased overall survival rate of cancer patients [49]. However, there are also side effects associated with CPD usages, such as myelosupression [20], cognitive dysfunction [50] and others [51]. The damage of CPD on bone was first reported in 1982, which indicated that the local administrations of CPD inhibited the mandibular growth and induced bone destructions in rats [52]. The accumulating clinical evidences has shown that the usages of CPD and its regiments caused osteoporotic phenotype in patients with breast cancer [10, 11, 53, 54]. Moreover, recent studies have indicated that the treatment of CPD in patients appear to suppress the expression of interleukin (IL)-6 [55], one bone resorption marker, and inhibit the osteoclasts-covered bone surface [6, 43]. Moreover, low dosage of metronomic CPD alleviated breast cancer-induced osteolysis [56]. Interestingly, the application of CPD caused bone loss but meanwhile increased the higher level of Osteocalcin and OPG in breast cancer patients [12]. Taken together, these studies indicates CPD may exert inhibits bone resorption by inhibits the activity of osteoclast. This is also our propose to address the biological influences and mechanism of CPD on bone, particular on osteoclast, are far more complicated than we have realized.

Previous clinical finding indicated that the application of CPD induced early onset of menopausal in women as well as bone loss in female mice [8]. Herein we studied the effects of CPD on bone biology of male mice. This study showed that CPD treatment induced the phenotype of bone loss in male mice, meanwhile, subsequently FACS assays in our study with CPD-treatment mice indicated that CPD also caused the decrease of MSC numbers. The reduced number of MSCs is one biological manifestation of CPD-induced microenvironment for bone metastasis [57], which is in accordance with the previous findings indicating that CPD-induced microenvironment promoted subcutaneous tumor growth and skeletal metastasis in animal models [58, 59].

CPD induces the expressions of several factors, such as C-C chemokine ligand (CCL) 2, IL-6, and VEGF-A, in bone marrow to create a pro-metastatic microenvironment for cancer cells and osteoclast to survive and the suppressed expression of MSCs in bone marrow. Therefore, the following bone mineral density (BMD) decreases are associated because of the deficiency of MSCs and accelerating osteoclast-induced bone loss that caused by bone metastasis [60]. The FACS result in this study indicated that CPD-treatment induced decreased numbers of MSCs in mice, which may contribute to the loss of bone mass. The decrease of osteoblastogenesis was further identified by ALP staining. CPD-induced decrease of bone formation and osteoblast activity were demonstrated by the down-regulation of the expression in osteoblastic differentiated factors, such as Runx2, Osteocalcin, col-1, Wnt 1, Wnt 4, Wnt10b and Cyclin D1 and the up-regulation of the expression of DKK1 in primary bone MSCs.

Previous investigations have indicated that CPD induced more adipogenesis rather that osteoblastogenesis by altering the switch of MSCs differentiation [21]. Studies have demonstrated that the over-activity of Wnt/β-catenin in MSCs changed the balance of adipocyte/osteoblast by inducing the osteogenesis transcription factors but inhibiting the adipogenesis transcriptions factors PPARγ, thus, induced more osteogenesis (ALP) and bone formation [61]. Therefore, the down-regulated expression of Wnt/β-catenin may account for CPD-induced bone loss because of the promotion of adipocytes instead of osteoblasts besides the direct inhibitory effects on osteoblast-mediated bone formation [13, 21]. This study indicated that CPD induced bone loss in bone remodeling by decreasing the number of MSCs, inhibiting osteoblastogenesis, and contributing to the suppressed bone formation and bone loss.

On the other hand, the molecular mechanism of CPD in RANKL-mediated osteoclastogenesis need to be further addressed. Although previous CPD study demonstrated that regiments CEF enhanced bone resorption [8, 54], CPD intervention suppressed bone resorption marker, such as IL-6 [55], and exerted bone protective effects on breast cancer-induced osteolysis [56]. M-CSF and RANKL are two necessary cytokines for osteoclastogenesis of BMMs [62]. M-CSF mainly promotes the survive and proliferation of BMMs, while RANKL drives BMMs into the osteocalst lineages commitment [34] and RANKL-activated signaling plays critical roles for the differentiation, survival, activity of osteoclast and consequently bone resorption [63, 64]. Therefore, the identification of the effects of CPD on RANKL-activated signaling will aid us to understand the systematic roles of CPD on osteoclastogenesis and bone remodeling. This study demonstrated the inhibitory effects of CPD on osteoclastogenesis, and the potential mechanism may be attributed to its suppressive effects of CPD on the expression of TRAF-6, NFATc1, NF-kB and c-Fos which are involved in RANKL-mediated osteoclastogenesis [38]. Meanwhile, CPD suppressed bone resorption by inhibiting osteoclast genes encoding MMP9, Ctsk, TRAP and Car2. The similar bone-protective effects of chemotherapy agents was identified by using one 2-methosyestradiol analogue ENMD-1198, which inhibited osteolysis in breast cancer-induced mice and exerted more pronounced bone-protective effects than low-dose metronomic CPD in mice model [56].

The inhibition of CPD on osteoclastogenesis was not in accordance with the findings that CPD led to osteoporosis phenotype in this study. Similar result was reported that thiazolidinedione (TZD), targeting PPARγ, exerted the inhibitory effects on both osteoclastogenesis and osteoblastogenesis, and finally induced bone loss and increased higher risk of fracture in patients with type 2 diabetes [65, 66]. As we all know, normal bone marrow is one complex microenvironment that houses hematopoietic stem cells (HSCs) and MSCs to maintain the balance of bone formation and bone resorption [45, 67]. It was supposed that even the inhibitory effect of CPD on bone resorption can partly compensate for bone loss mainly caused by the influences of CPD on MSCs and osteoblastogenesis in this study, it cannot fully keep up with the rapid bone loss. Hereby, our study potentially indicated that CPD inhibited bone formation and bone resorption in favor of bone formation. Therefore, the detailed regulatory mechanism of CPD on bone remodeling still need to be further investigated.

For clinical practice, this study indicates that the bone formation plays crucial roles in preventing CPD-induced bone loss. The treatment for bone loss induced by marketed drugs in treating chemotherapy are mainly targeting osteoclast to inhibit bone resorption [68], but the clinical effect is not good as expected. Our findings demonstrated that targeting osteoblasts to improve osteoblastogenesis might be one effective approach, and the novel biochemical agents, stimulating osteoblastogenesis and suppressing adipogenesis, may exert better bone-protective effects in rescuing CPD-induced bone loss.

The usages of CPD have been reported to cause side effects on a number of organs. Studies have reported that CPD caused hepatoxicity, nephrotoxicity, reprotoxicity, cardiotoxicity, and myelosuppression [69–71], which may account for bone loss by their indiect effects on bone:1). The reprotoxicity accelerates bone loss by decreasing the sexual hormones such as estrogen and androgen [10]; 2). The nephrotoxicity lead to the disorder of the metabolism of calcium and phosphorus and consequently bone loss. But the direct effects of CPD on bone remodeling are still not fully understood. Our study first established the direct effects of CPD on bone remodeling by both affecting osteoblastogenesis and osteoclastogenesis. Furthermore, our study also support that CPD caused myelosuppression as shown by decreased the number of bone MSCs in CPD treated mice (Figure 4A and 4B). At the molecular level, the inhibitory effect of CPD is similar to the inhibitory effects of methotrexate, one chemotherapy agent, that exert on the expression on Wnt/β-catenin signaling in bone [72].

The biological influences of CPD on bones have closely relationship with the dosages, and frequency of the interventions while the proper selection for CPD can produce less toxicity/less side effects for both animal and *in vitro* investigation. Normally, the severity of toxicity to normal cells in humans and experimental animals is increased with the high dose of CPD or CPD regiments. According to the dosages in clinical application, the dosage of CPD and CPD regiment ranged from 10 mg/kg to 350 mg/kg but with different cycles of chemotherapy treatment. One study induced bone disorders by intravenous tail injections of CPD regiment (10 mg/kg) for six cycles (weeks) in Sprague-Dawley rats [18], indicating that the low dosages of CPD also cause toxicity on bone tissue. Moreover, commercial CPD was administrated (50mg/kg) with intraperitoneally to Swiss albino mice to induce the hypercellularity in bone marrow and spleen [73]. Furthermore, a single dosage of CPD (150mg/kg) induced rapid and severe hematopoietic suppression in male Ku Ming (KM) mice [20] and same dosages of CPD were performed to induce the myelosupression and bone osteoporosis phenotypes in C57BL/6 mice [74]. In addition, the intraperitoneal injection with the one single dosage of CPD (200mg/kg) has been reported to cause the myelosupression in male BALB mice [75]. To our knowledge, the highest dosage (350 mg/kg, i.p) intervention with of CPD has been identified to enhance experimental prostate cancer metastasis [76]. In our study, we treated the mice with 100mg/kg of CPD at 7 seven consecutive interventions. Our intervention was able to induce commonly seen side effects, such as myelosupression, based on the previous animal study and thereafter to investigate their effects on bone loss in C57BL/6 male mice. However, the dosage and intervention time was performed at one rather moderate level but not at lethally dosage. Meanwhile, CPD at 2000 μM (CPD-D) could effectively inhibit osteoclastogenesis (Figure 6F and 6G) but exerted no significant effects on cell viability of BMMs (Supplementary Figure 2). Therefore, these data indicated that the osteoclastogenesis inhibited by CPD was not due to the toxicity of CPD on BMMs.

However, there are still several limitations in our studies. First, our study was performed with male mice and the exact effects of CPD on female have not been investigated based on our preliminary finding indicates that the male mice were more sensitive than female for CPD intervention [77]. Therefore, future work will be performed to address this issue. Second, CPD are commonly prescribed with regiments, therefore, it is necessary to carry out more investigations on CPD regiments. Third, the intervention time with CPD is short (7 days). Finally, only one dosage was performed in this *in vivo* study and the dose-dependent effect of should be studied. Taken together, the dosage, and frequency is vary to the clinical practice, and will limit the usages in the management of patient.

In summary, our animal and *in vitro* studies have demonstrated the following points: 1) CPD treatment induced the osteoporotic phenotype in mice associated with the decrease of bone formation and resorption. 2) CPD exerted inhibitory effects on osteoblastogenesis by suppressing Wnt/β-catenin signaling. 3) CPD exerted inhibitory effects on RANKL-mediated osteoclastogenesis by suppressing RANKL-activated signaling. 4) The changes of bone formation, other than bone resorption, played critical roles in CPD-induced bone loss.

## MATERIALS AND METHODS

### Reagents

Cyclophosphamide (CPD) was purchased from Shengdi Company (Jiangsu, China). M-CSF and RANKL were purchased from Peprotech (New Jersey, USA). Primary antibodies against active β-catenin, Runx2, Cyclin D1, c-Myc, IκBα, phospho-IκBα, JNK, phospho-JNK, p38 and phospho-p38 were purchased from Cell Signaling Technology (Beverly, MA, USA) and others against TRAF-6, TRAF-3, NFATc1, RANK and c-Fos were purchased from Santa Cruz Biotechnology (CA, USA). The antibodies against β-catenin, phospho-Gsk-3β, and DKK1 were purchased from Abcam Company (Cambridge, UK).

### Animal treatments

Six-week-old male C57BL/6 mice (SLAC Laboratory Animal Co. Ltd., Shanghai, China) were maintained in animal center of Longhua hospital with light: dark (12h:12h) condition and fed with commercial diet and distilled water ad libitum during experimental period. The mice were administrated with CPD (100mg/kg/day) by intraperitoneal injection for 7 consecutive days and subsequently maintained for additional 3, 7, and 12ds. For each time points, tibia and lumbar vertebra 1~3 were collected for histological analysis and lumbar vertebra 4~5 were harvested for RNA analysis. All of the experimental protocols were performed with the approval of Institutional Animal Care and Use Committee of the Shanghai University of Traditional Chinese Medicine, and conformed to the National Institutes of Health Guide for Care and Use of Laboratory Animals (Publication No. 85-23, revised 1985).

### μCT analysis

Tibia and LV1~3 were fixed in 10% Neutral Buffered Formalin (Wexis, Guangzhou, China) for 24h, washed for 2 hours with tap water, and examined. The samples in each group were scanned to develop three-dimensional (3D) mode. The analyses were carried out using μCT80 radiograph microtomograph (Scanco Medical AG, Switzerland), associated with 3D reconstruction by μCT Ray V3.4A model visualization software (Scanco Medical AG, Switzerland). The following quantitative parameters including bone mineral density (BMD), ratio of bone volume to tissue volume (BV/TV), trabecular bone numbers (Tb.N), trabecular bone thickness (Tb.Th), and trabecular separation (Tb.Sp), were obtained.

### HE staining

Tibia and lumbar vertebra 1 ~ 3 were fixed in 4% paraformaldehyde for 24 hs, decalcified in 10% EDTA for 4 weeks, and embedded in paraffin wax. The sections were stained with hematoxylin and eosin (H&E). Subsequently, the representative images were obtained with auto capture system (Olympus BX50, Tokyo, Japan).

### Immunohistochemistry analysis

Tibia sections (5μm) were deparaffinized, rehydrated with PBS (pH 7.4), treated with aqueous 3% H_2_O_2_ for 10 min, and antigen was retrieved with 0.1% trypsin (M/V) in 0.1% CaCl_2_ at 37°C for 10 min. The sections were blocked with 5% BSA at RT for 30min, then the antibodies for active β-catenin, Runx2, Osteocalcin, TRAF-6, NFATc1, c-Fos, MMP9, and Ctsk were applied at the concentration of 1:200 and incubated overnight at 4°C. The slides were incubated with second antibody conjugated HRP for 15 minutes after washing with PBS. Diaminobenzidine was applied for 5 minutes, and slides were then counterstained with H&E, dehydrated, and mounted with cytoseal mounting medium. The images were obtained by the imagine auto analysis system (Olympus BX50). Bone morphometric analysis was performed with an image autoanalysis system (Olympus BX50, Tokyo, Japan)

### Flow cytometry analysis

Flow cytometry antibodies anti-CD29-FITC, and CompBeads were purchased from BD biosciences (New Jersey, USA). Anti-CD45-PerCPcy 5.5 was from Biolegend (USA) and anti-CD44-APC was from eBioscence (SanDiego, CA, USA). Bone marrow cells from tibia and femur of 10-12-week-old saline or CPD-treated (7 consective days, dosages) male mice were flushed out with PBS plus 2.5% Fetal bove serum (FBS), filtered, centrifuged, removed the red blood cell, and collected. The final concentrations were 1×10^7^/ml before performing flow cytometry assays. The negative expression of CD45-PerCPcy5.5, and positive expression of CD29-FITC, and CD44-APC were obtained and analyzed with BD FACS Aria III (New Jersey, USA), and one representative assay was shown.

### CM-CPD working solution preparation and identification

CPD working solution study was prepared with conditioned medium (CM) for *in vitro* investigations as described previously [78]. Briefly, the mouse hepatocyte BNLCL.2 cells (Stem Cell Bank from Chinese Academy of Sciences, Shanghai, China) were cultured in α-MEM containing 10% FBS and 1% P-S to 70% confluent before starving in α-MEM with 1% P-S for 12 h. CPD with the concentrations of 4000μM was added to the α-MEM containing 2% FBS, 1% P-S and subsequently cultured for 24 h. The supernatants were centrifuged and collected before filtering with 0.22 μm filter unit and are prepared as the primary CM-CPD working solution.

To identify the efficacy and quality of CM-CPD working solution, the liquid chromatography mass spectrometry (LC-MS/MS) analysis were performed. The standard curve (nanogram-peak area) was made with 2 time's serial dilution of 4-Hydroperoxy CPD (from 109ng) which easily converted to 4-OH-CPD. In brief,10μL standard dilution (plus 190μL CPD-Control), or 200μL CPD (4000μM) was mixed immediately with 20μL of semicarbazide (SCZ; 2M) to derivative 4-OH-CPD into 4-OH-CPD-SCZ and was vortexed before placing in a water-bath at 35°C for 2 h. Next, 100μL acetonitrile was centrifuged, and the supernatant was extracted twice with ethyl acetate. The organic phase was evaporated to dryness and reconstituted with mobile phase (water-acetonitrile, 50:50, and vol/vol). The LC separation was performed on a Thermo Scientific Hypersil Gold column with water-acetonitrile (90:10, vol/vol) as the starting gradient, at a flow-rate of 0.3mL/min with a total run time of 15 min. Mass transitions were monitored at m/z: 334→221 for 4-OH-CPD-SCZ under the positive multiple reaction monitoring (MRM) mode. The curves in consistence of 4-OH-CPD-SCZ are regarded as meet the quality control and selected to perform *in vitro* study (Figure 1).

Different concentrations of CM-CPD working solutions were prepared with proper dilutions of the primary CM-CPD (4000 μM) working solution. For convenience, the working solutions with CPD at the concentration of 0 μM, 500 μM, 1000 μM, 1500 μM, 2000 μM, 2500 μM, 3000 μM,3500μM, 4000 μM were marked as CPD-Control (CPD-Con), CPD-A, CPD-B, CPD-C, CPD-D, CPD-E, CPD-F, CPD-G, and CPD-H respectively, through the whole *in vitro* study.

### ALP and alizarin red staining

For osteoblast differentiation assays, the primary mesenchymal stem cells (MSCs) were isolated from the bilateral femur and tibia of naïve, saline control or CPD-treated (100mg/kg/day) male C57BL/6 mice. Bone marrow cavity was flushed with α-MEM (Hyclone laboratories, Utah, USA) containing 10% fetal bovine serum (FBS, Gibco, Carlsbad, CA, USA) and 1% penicillin-streptomycin. MSCs were cultured with osteoblast differentiation introduction medium (α-MEM complete medium containing 10mmol/L β-Glycerophosphate (Sigma), 50mg/L L-ascorbic acid (Sigma), 10^-8^mol/L Dexamethasone (Sigma)) for 7ds with media refreshed twice weekly. Then cells were fixed for 20min with 10% Neutral Buffered Formalin followed by washing twice with PBS. ALP staining reagent 1-Step NBT-BCIP (Pierce, Rockford, IL, USA) were added to wells of 24-well plate for 0.5-1h at 37°C and the positive staining was shown by Lyons blue. MSCs from naïve mice were cultured with CPD-Con, CPD-B, CPD-D, and CPD-H for 3 days before cultured in the osteoblast differentiation introduction medium for ALP staining or Alizarin red staining. To examine the capacity of mineralized nodule formation, prolonged osteoblast differentiation induction was performed until 21ds with media refreshed twice weekly. The cells were fixed and washed as the ALP staining, then were stained with 1% alizarin red at 37°C for 30 min, washed for observation.

### TRAP staining

Bone marrow macrophages (BMMs) were isolated from the bilateral femur and tibia from normal or CPD-treated (100mg/kg/day) male C57BL/6 mice, seeded per well with 5×10^4^ cells in 24-well tissue treated plates and treated with M-CSF (44 ng/ml) and RANKL (44 ng/ml) for 5 days to generates osteoclasts. Matured osteoclasts were identified with TRAP staining by commercial kit (Sigma). TRAP-positive cells with multi nuclei (>3) are considered as osteoclast. The images were obtained using Leica DMI300B Microscope (Solms, Germany).

For *in vivo* study, TRAP staining was performed on paraffin sections of tibia. Briefly, the sections were deparaffinized, and dehydrated with 0.02g/ml Napthol-Ether solution which was diluted with basic stock solution at the ratio of 1:50 for 1 h. The sections were dehydrated and incubated with the diluted Napthol-Ether solution at 37°C for 30 minutes. Then a 0.04g/ml sodium nitrite solution and a 0.05 g/ml pararosaniline dye/2 N hydrochloric acid were mixed before adding to the basic stock solution. The sections were incubated in the mixture for additional 15 minutes. After counter staining and dehydration, the sections were mounted and observed using light microscope (Olympus BX50) and image analysis was performed with software mentioned previously [79]. TRAP activity was quantified by calculating the ratio of the number of TRAP-positive osteoclasts in each representative view area [80].

### MTT assay

Cell viability was analyzed by 3-(4, 5-dimethy-lthiazol-2-yl)-2,5- Diphenyltetra-zolium bromide (MTT). Briefly, MSCs were seeded at a density of 2×10^4^ cells with sextuplicate in 96-well plates. Primary bone MSCs were treated with CPD-Con, CPD-B, CPD-D and CPD-H for 0h,24h,48h,72h, while BMMs were treated CPD working solutions (Control, CPD-Con,CPD-A to CPD-H for 5ds before adding 20 μl MTT (5 mg/ml) to each well. The cells were cultured at 37°C for 4 h and the supernatant was discarded. Subsequently, 200 μl DMSO was added and the absorbance was read at 570 nm using a Varioskan TM Flash Multimode Microplate Reader (Thermo Scientific, Waltham, MA, USA). Each assay was independently repeated 3 times and the percentage of cell viability was calculated according to OD ratio. Cell viability (%)=OD _treated_/OD _control_ ×100%.

### Western blot assays

MSCs were isolated from C57BL/6 mice on day 3 after 7 doses of CPD at 100mg/kg injection daily were obtained as plastic adherent cells and subculture to passage 3. MSCs or BMMs isolated from naïve C57BL/6 mice were cultured for adherent, and passage 3 cells in 10cm dishes of 75% confluent were treated with CPD-H for different times. C3H10T1/2 was treated with CPD-H in 10cm dishes for different times. The total protein were extracted using the RIPA lysis buffer (Beyotime, Shanghai, China) supplement with 1% protease inhibitors (Beyotime, Shanghai, China) and 1% phosphatase inhibitors (Sigma-Aldrich, St Louis, USA). The cytoplasmic protein, and nuclear protein were extracted from C3H10T1/2 treated with CPD-H for different times using the NE-PER Nuclear and Cytoplasmic Extraction kits according to the manufacturers’ instructions.

The protein lysates from bone primary cells or *in vivo* experiments were subjected to western blot analysis as described previously [7, 76]. The primary antibodies (Cell signaling, MA, USA) against TRAF-3, TRAF-6, NFATc1, RANK, c-Fos, active β-catenin, Runx2, Cyclin D1, c-Myc, Gsk-3β, phospho-GSK-3β and DKK1 were incubated in 5% non-fat dry milk solution (TBS containing 0.1% Tween 20). Membranes were washed extensively, and an ECL detection assay was performed using a Super Signal West Dura kit (Beyotime, Shanghai, China).

### Real-time RT-PCR

Lumbar vertebrae of mice after 7 doses of CPD at 100mg/kg injection daily were separated and detached cleanly in 2ml eppendorf tubes containing 1ml TRIzol and 3 steel balls, grinding in Tissuelyser-24 according to the manufacturer's instructions. C3H10T1/2 was treated with CPD-H in 6-well plate for different times. Total RNA was prepared using TRIzol reagent (Invitrogen, Mulgrave, Australia) according to the manufacturer's instructions. First-strand cDNA was synthesized from 1μg of total RNA by incubating for 15min at 37°C with Prime Script RT reagent Kit (Takara) following oligo(dT) priming. After reverse transcription reaction, qRT-PCR was performed by CFX96 PCR system (Bio-Rad) using SYBR1Premix Ex Taq^™^ (Takara, Dalian, China) according to the manufacturer's instructions. Data were analyzed using the comparison Ct (2^-ΔΔCt^) method, and expressed as fold change compared with respective control. Each sample was analyzed in triplicate and genes expression of Osteocalcin, ALP, Runx2, Wnt1/4/10b, β-catenin and Cyclin D1 were normalized to the housekeeping gene β-actin. The primer sequences for the genes were shown in Table 1.

**Table 1:** The sequences of primers used in this study

ß-actin:
forward,5′-CCTGTACGCCAACACAGTGC-3′;
reverse, 5′- ATACTCCTGCTTGCTGATCC-3′;
Oc:
forward, 5′- CTTGAAGACCGCCTACAAAC-3′;
reverse, 5′- GCTGCTGTGACATCCATAC-3′;
ALP:
forward, 5′- GAATCAAATGTTCAGGGTGGT-3′;
reverse, 5′- TGGCACGTTAAAGGTAATCAG-3′;
Runx2:
forward,5′-CTCTTCTGGAGCCGTTTATGT-3′;
reverse, 5′- GTTTCTTAGGGTCTTGGAGTGA-3′;
Wnt1:
forward, 5′- ACAGCGTTCATCTTCGCAATCACC-3′;
reverse,5′-AAATCGATGTTGTCACTGCAGCCC-3′;
Wnt4:
forward, 5′- CTCAAAGGCCTGATCCAGAG-3′;
reverse,5′- GTCCCTTGTGTCACCACCTT-3′;
Wnt10b:
forward, 5′- TAACCACGACATGGACTTCGG-3′;
reverse,5′-TCCGCTTCAGGTTTTCCGTTA-3′;
Cyclin D1:
forward,5′-CTCCGTATCTTACTTCAAGTGCG-3′;
reverse, 5′- CTTCTCGGCAGTCAAGGGAA-3′;
ß-Catenin:
forward,5′-CACGCAAGAGCAAGTAGCTG-3′;
reverse,5′-TCTGTGATGGTTCAGCCAAG-3′;

### Semi-quantitative PCR analysis

Total RNA was isolated from BMMs with TRIzol reagent (Invitrogen, Carlsbad, CA, USA). Superscript III transcriptase (Invitrogen) was used to perform the reverse transcription assays according to the standard protocol. The synthesized cDNA in a 20 μl volume was applied for regular PCR using Arktik thermal cycler (Thermo Scientific, Vantaa, Finland). The expression of matrix metalloproteinase 9 (MMP9), cathepsin K (Ctsk), tartrate-resistant acid phosphates (TRAP) and carbonic anhydrase II (Car2) and GAPDH genes was measured as previously described [79]. The PCR products were loaded on 2% agarose gel for electrophoresis analysis. Images of the targeted bands were obtained using GIS-2009 (Tanon Innotech Corporation, Beijing, China).

### Statistical analysis

The GraphPad Prism 5.0 software (GraphPad Software, Inc.) or Excel (2007) was used for the statistical analyses. The results were expressed as mean ± S.D under each independent condition. Statistical analyses between two groups were assessed by unpaired two-tailed Student's t test. Statistical analyses among multiple groups were carried out using ANOVA with Bonferroni adjustment. *P* values less than 0.05 are considered statistically significance.

## SUPPLEMENTARY MATERIALS FIGURES


