# Supplementary material for: Cyclophosphamide causes osteoporosis in C57BL/6 male mice: suppressive effects of cyclophosphamide on osteoblastogenesis and osteoclastogenesis
Source: Oncotarget. 2017 Sep 18;8(58):98163–83. doi: 10.18632/oncotarget.21000 (PMC5716721; doi:10.18632/oncotarget.21000)
Supplement: Supplementary file 1 [file oncotarget-08-98163-s001.pdf]

Cyclophosphamide causes osteoporosis in C57BL/6 male mice: suppressive effects of cyclophosphamide on osteoblastogenesis and osteoclastogenesis

SUPPLEMENTARY MATERIALS

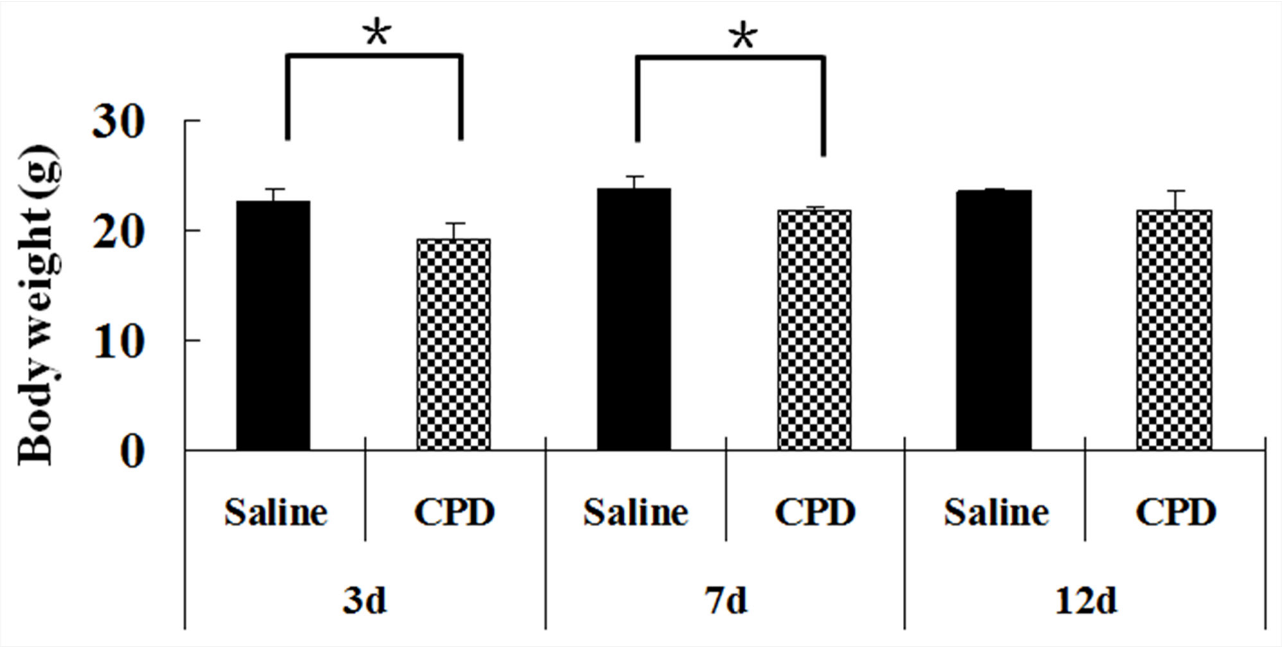

Supplementary Figure 1: Cyclophosphamide induces body weight loss at 3d and 7d but not at 12d. Male C57BL/6 mice were administered with saline or cyclophosphamide (CPD, 100mg/kg/days (d)) for 7 consecutive days by intraperitoneal injections. The mice were maintained for additional 3,7and 12 days and the body weight were assessed at each time point.

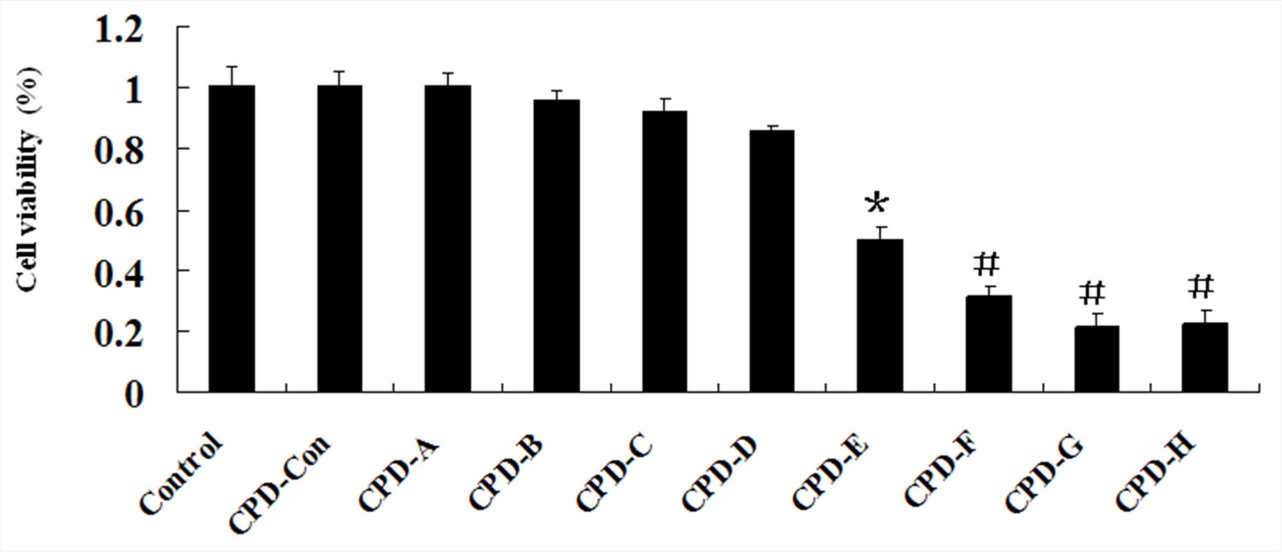

Supplementary Figure 2: Cyclophosphamide (CPD) did not affect the cell viability of bone marrow macrophages (BMMs). BMMs were treated with M-CSF (44 ng/ml), or M-CSF (44 ng/ml) and CPD-Con(0 μM), CPD-A(500 μM), CPD-B (1000 μM), CPD-C(1500 μM), CPD-D (2000 μM), CPD-E(2500 μM), CPD-F (3000 μM), CPD-G (3500 μM), CPD-H (4000 μM) for 4 days. The culture was performed with the MTT assays according to the standard protocol. All assays were performed with triplicate and independent repeated for 3 times, and one representative set of assays was shown. $^{*}P < 0.05$, $^{\#}P < 0.01$ vs. control.
